# Associations Between Self, Peer, and Teacher Reports of Victimization and Social Skills in School in Children With Language Disorders

**DOI:** 10.3389/fpsyg.2021.718110

**Published:** 2021-11-15

**Authors:** Inmaculada Sureda-Garcia, Mario Valera-Pozo, Victor Sanchez-Azanza, Daniel Adrover-Roig, Eva Aguilar-Mediavilla

**Affiliations:** Department of Applied Pedagogy and Educational Psychology, Institute of Research and Innovation in Education, University of the Balearic Islands, Palma, Spain

**Keywords:** developmental language disorder (DLD), reading difficulties (RD), victimization, bullying, sociogram, teacher report, peer-rejection

## Abstract

Previous studies have shown that teachers and parents of children with language disorders report them to have higher victimization scores, a heightened risk of low-quality friendships and social difficulties, and may be more vulnerable to peer rejection than control peers. However, there are few studies of bullying in children with developmental language disorder (DLD) and reading difficulties (RD), and none has considered the mutual relationships between teacher reports, the perceptions of classmates, and children’s self-reports. We analyzed the experiences of bullying and peer relationships in primary school students with DLD and RD as compared to their age-matched peers using teacher reports, peer reports, and self-reports on victimization. Additionally, we explored how these three perspectives are associated. Results indicated lower levels of peer-rated prosocial skills in DLD and RD students compared to their peers, as well as higher levels of victimization as assessed by peers for students with DLD. In the same line, the teachers’ ratings showed that students with DLD presented poorer social skills, less adaptability, and more withdrawal in social interaction. Contrastingly, self-reports informed of similar rates of interpersonal relationships, social stress, and peer victimization between the three groups. Consequently, we found significant correlations between measures of peer reports and teacher reports that contrasted with the lack of correlations between self and other agents’ reports. These findings stress the importance of using self-reports, peer reports, and teacher reports at the same time to detect bullying situations that might go unnoticed.

## Introduction

Connecting with others is fundamental in childhood and adolescence, as students are continuously seeking for support, liking and acceptance from their peers. Peer relationships help to develop socio-emotional skills, cope with life challenges, and reduce stress and anxiety. Thus, social interaction and close relationships are important for both physical and mental health (Adriaensens et al., 2017). Successful relationships require adequate communication skills, as these skills help to respectfully interact and discuss with others, reducing the stress in social conflicts and allowing to achieve better solutions in these situations (Pipaş and Jaradat, 2010). In addition, communication skills allow to better understand other people, reducing the appearance of misunderstandings and frustration (Erozkan, 2013). In this regard, difficulties in communication skills of children with language disorders (oral or written) may be a common source of these problems and social deficits (Mok et al., 2014). Consequently, children with developmental language disorder (DLD) and reading difficulties (RD) can experience problems with peer relationships during childhood, and especially throughout adolescence (Mok et al., 2014; Forrest et al., 2021; Ibáñez-Rodríguez et al., 2021). Both groups (DLD and RD) have in common a language difficulty that could affect reading but, following the Simple View of Reading (Hoover and Gough, 1990), the primary difficulty of children with DLD concerns oral language (and not decoding in transparent languages; Buil-Legaz et al., 2015), whereas children with RD have a deficit in their phonological abilities that leads to difficulties in decoding. Both aspects, decoding and oral language, are crucial in the schooling process, and effects could lead to learning disabilities and social inadaptations. The study of both groups can help to elucidate how these variables affect socialization at school.

DLD, formerly known as specific language impairment (SLI), is a persistent language delay affecting communication and/or learning, in the absence of biological, cognitive or psychological conditions (Bishop et al., 2016, Bishop et al., 2017). In addition to language difficulties, children and adolescents with DLD have difficulties in social relationships, such as poor acceptance from their classmates, low quality and quantity of friendships, and higher rates of peer rejection (Laws et al., 2012; Mok et al., 2014; Lloyd-Esenkaya et al., 2020). Therefore, students with DLD of different ages show more peer problems than typically developing students (Lindsay and Dockrell, 2012; Levickis et al., 2018). These social difficulties increase from childhood to adolescence, as these are less prevalent in young children (Levickis et al., 2018). More specifically, these complications reach their maximum at age 16, when they can be up to 5 times more predominant than in typically developing youngsters (Lindsay and Dockrell, 2012; Van den Bedem et al., 2018a).

RD is the most prevalent type of learning disability, with a prevalence between 7 and 10% (Bhakta et al., 2002; Carrillo et al., 2011; Cecilia et al., 2014), depending on the specific difficulty measured (low speed and/or accuracy rate). It includes impairments in reading decoding (i.e., letter-phoneme correspondence) resulting from problems in phonological processing skills and/or naming problems (Ramus et al., 2013; Smith-Spark et al., 2017). Children with RD also show impaired oral language skills, although not as severe as children with DLD (Goulandris et al., 2000; Bishop and Snowling, 2004; Ramus et al., 2013). Similar to children with DLD, parents, teachers, and peers have a negative perception about social issues of children with RD (Undheim et al., 2011; Yildiz et al., 2012). This social negative perception can affect children with RD, lowering self-esteem, and causing behavioral problems and social anxiety (Sako, 2016). Moreover, findings on students with RD report that they are less socially competent, use maladaptive strategies (such as withdrawal or aggressiveness) more frequently than their normative peers, and tend to feel excluded at school (Undheim et al., 2011; Turunen et al., 2017). The abovementioned problems can also persist during adulthood (Ghisi et al., 2016).

As there is evidence that children and adolescents with DLD and RD have poorer friendships, lower acceptance from their peers and even deficits in social cognition, they are more likely to suffer victimization or bullying (Humphrey and Mullins, 2004; Redmond, 2011). Bullying can be defined as a type of intentional and systematic interpersonal violence, inflicted by one or more children toward another who is in a situation of inequality and becomes a victim (Olweus, 2013). Thus, peer relationships become unbalanced and regulated by the domination-submission schema. This schema represents an asymmetric relationship, in which bullies take advantage of their power over the victims, who do not feel able to stop the aggressions. In this way, aggressors obtain control and power over the victim. Consequently, bullying seems to inhibit the victims’ social interactions leading to social rejection, exclusion, and victimization (Monjas et al., 2014).

Some researchers (Knox and Conti-Ramsden, 2003, 2007; Redmond, 2011; Laws et al., 2012) have reported that children and adolescents with DLD are up to 3 times more likely to suffer bullying than typically developing peers. However, other studies (Conti-Ramsden and Botting, 2004) did not find a significant correlation between social variables and overall language scores, the former being mainly related to a deficit in pragmatics. Thus, it is difficult to relate social problems experienced by children and adolescents with DLD, victimization and the nature of their linguistic impairment. The severity of DLD, in terms of the level of impairment in several language components such as grammar, vocabulary, and pragmatics using standardized tests, seems to explain only a small part of the variance reported in victimization (Andrés-Roqueta et al., 2016), which suggests that other factors, such as social skills, social cognition, and emotional competence (Van den Bedem et al., 2018a), might be also involved. Nevertheless, another study (van der Wilt et al., 2018) has revealed a relationship between language deficits and social issues as those abovementioned. Specifically, children classified by their peers as rejected or neglected with a sociogram showed lower oral communicative skills (communicative functioning and conversational skills) than their popular or average classmates. In brief, although the previous literature shows mixed results on the relation between some of the language problems of DLD and a host of social variables, the latest reports indicate the interplay of these aspects to some degree. Thus, further exploring this association seems worthy to discern whether language difficulties are related to social (and, likely, victimization) problems in children with DLD.

Related to this, several authors have also found higher victimization rates in children and adolescents with RD than in their normative peers (Humphrey and Mullins, 2004; Turunen et al., 2017). Boyer et al. (2019) reported that bullying victimization correlates with internalizing problems in children with dyslexia. Moreover, Turunen et al. (2017) associated RD with involvement in bullying as victims, bullies, and bully/victims. In the same study, these authors related a lower social self-concept to victimization, aggressive behavior and poorer school adjustment in children with RD. Therefore, lower self-concept, self-esteem and negative feelings when compared with others might explain social problems in children and adolescents with RD, which can lead to be involved in bullying situations. Although children with RD also show language difficulties, none of the previous studies has explored the role of their language problems on bulling and peer relationships.

Different explanations for these outcomes can be associated with the potentially higher risk of social difficulties and bullying victimization in children with DLD and RD, which could also explain individual differences between them. First, they can be more rejected due to their language and communicative deficits (Fujiki et al., 2013; Andrés-Roqueta et al., 2016; Sako, 2016). Second, this rejection can appear as a result of difficulties in emotional understanding due to their poorer language skills (Lloyd-Esenkaya et al., 2021). Third, they can present deficits in social cognition and lower adequacy in communicative situations (Conti-Ramsden and Botting, 2014; Bakopoulou and Dockrell, 2016; Font-Jordà et al., 2018; Valera-Pozo et al., 2020).

Nevertheless, not all children with DLD and RD show peer problems and victimization. According to Brinton et al. (2000), the success of social interactions in students with DLD and RD was highly variable, as some children with DLD presented higher levels of aggressive or withdrawing behavior, while some others showed a typical social profile (Lloyd-Esenkaya et al., 2020). Furthermore, a recent study (Ibáñez-Rodríguez et al., 2021) reported that children with DLD do not present higher victimization than typically developing peers in a global measure of victimization, but they are more victimized when bullying victimization scores are specifically related to language reasons (for example, mockery because of language mistakes or misunderstandings). In this vein, Mok et al. (2014) reported a moderate percentage of children (22.2%) with few or no problems in peer relationships, and Fujiki et al. (2001) did not find higher victimization in children with DLD than in typically developing children.

In addition to the heterogeneity in these profiles, there is another important source of controversy that involves the perception by different agents in social settings. Several of the previously mentioned studies are based on ratings by teachers, parents and/or classmates, who frequently share similar opinions. Previous works (Graham and Juvonen, 1998; Bouman et al., 2012) have reported that, while peer reports of victimization are more associated with perceived popularity, likeability and rejection, self-reports of victimization are usually stronger predictors of internalizing problems or intrapersonal consequences of victimization. Since peer reports do not seem to add unique variance when self-reports are considered (Bouman et al., 2012), peer and self-reports are regarded as two different measures, representing complementary perspectives on bullying (Hawker and Boulton, 2000). Thus, as outcomes of peer and self-reports correlate only moderately, there is a risk of not identifying all children involved in bullying using only one method (Berger and Rodkin, 2009; Gough Kenyon et al., 2021). Therefore, peer and self-reports provide distinct prevalence rates (Ladd and Kochenderfer-Ladd, 2002; Cook et al., 2010; Gough Kenyon et al., 2021). In light of this, children themselves might be better informants about their own feelings than other sources, and parents and teachers might detect behavioral symptoms better than children (Hankin and Abramson, 2001; Valera-Pozo et al., 2020). However, for the identification of victims both methods should be used because these identify different children, at least partially (Berger and Rodkin, 2009; Gough Kenyon et al., 2021).

In this respect, some studies have pointed out that many children and adolescents with DLD and RD rated their own social competence and relationships better than their peers and teachers did. The available data suggest that teachers and peers (and even parents) often judge the social abilities of individuals with DLD and RD as poor (maybe driven by their language deficit), while these children might have, in contrast, a more positive and maybe biased perception of their own social skills (Undheim et al., 2011; Wadman et al., 2011; Valera-Pozo et al., 2020; Gough Kenyon et al., 2021). Thus, it is plausible that biased self-perceptions of social abilities might extend to a biased interpretation of contextual social cues, which could support the model of social information processing (Crick and Dodge, 1994). However, it is also possible that other variables play a role in the divergence between self-and parent-reports, such as neuroticism. In this vein, parents of children with specific education needs might develop more symptoms associated with negative affect and neuroticism (emotional stability), in contrast to parents of typically developing children (Woodman, 2014). Higher levels of neuroticism promote maladaptive reactivity to stress, and difficulties in negative emotional regulation are known to be a predictor of several types of psychosocial self-reported problems (Schmitz et al., 2003; Lönnqvist et al., 2009). Parents with high neuroticism can show poorer parenting skills and a biased assessment of their children’s psychosocial characteristics, reporting more problems than parents with low neuroticism do (Ellenbogen and Hodgins, 2004; Koenig et al., 2010). Furthermore, children and adolescents who frequently experience negative emotions may be more strongly affected by the negative social experiences with peers or lack of social support that often result from socially withdrawn behavior (Smith et al., 2017). Cheng and Furnham (2020) indicate that parents with high levels of neuroticism (especially mothers) experienced more malaise and had a less happy relationship with their child, who expressed more behavioral problems. This, in turn, may have exaggerated their anxiety, depression and moodiness. Thus, it is plausible that other factors beyond the linguistic domain might play a role in the evaluation of victimization in children with DLD.

For these reasons, we explored the associations between the perceptions of teachers (in terms of social skills, adaptive behavior, and withdrawal), same class peers (related to prosocial behavior and victimization) as a sociometric approach, and the students themselves (self-reports on victimization, social skills, and adaptability) triangulating all outcomes to provide a more comprehensive account on the difficulties of children with DLD and RD. We expected that children with DLD and RD would be rated by their teachers as less socially skilled, having less adaptive behavior, and experiencing larger withdrawal in the scholar context. We also predicted that children with DLD and RD would show more victimization and less prosocial behavior scores and would be more victimized according to peer-reports. In addition, we expected that children with DLD and RD would rate themselves with lower victimization scores than peers and teachers would do. Finally, as peer and self-reports do not seem to evaluate the same constructs and correlate only moderately, we expected that reports carried out by other agents (i.e., peer and teacher reports) would show larger associations between them than those observed between self-and other agents’ reports.

## Materials and Methods

### Participants

This study is part of a larger longitudinal study with 114 participants. Children were recruited from 10 schools located in the Balearic Islands (Spain) and were in 4th, 5th, and 6th grade of primary education. All educational centers reported on cases of children with non-transient language difficulties affecting communication or learning [diagnosed with oral (DLD) or written and reading language difficulties (RD)]. For each participant with language difficulties, the speech and language therapist from the centers selected a same-class control participant matched for age and sex, with similar dominant language and social characteristics.

Departing from the diagnoses made by the school services, we verified that children met the criteria of DLD, RD or control following the Simple View of Reading model and the Catalise criteria (see Table 1). First, the speech-therapists of the schools answered a questionnaire asking about sociodemographic, medical conditions, developmental trajectory in the school, communication and learning history of the child. At the same time, we applied different tests (Core Language Score of the CELF-4, PROLEC subtests, Raven Progressive Matrices) to all children whose parents signed an informed consent (one dropped from the sample lacking the parents’ signature). In addition, the Balearic Department of Health conducts an Otoacoustic Emissions screening for all children attending Primary School. In this sense, none of the children presented either visual or auditory problems. Moreover, none of the children in the sample showed autistic traits, based on information provided by schools’ language therapists, nor mental disability, as measured by the Raven’s Progressive Matrices Test (IQ standard score greater than 75) (Raven et al., 1995).

**TABLE 1:** Data of participants.

Variables	Con	DLD	RD	Differences between groups Dunn’s *post hoc* or χ^2^		
	*M*(*SD*)	*M*(*SD*)	*M*(*SD*)	Con-DLD	Con-RD	DLD-RD
N	19	14	21			
Age (years; months)	10;10 (0;10)	11;2 (1;5)	11;0 (1;0)	*p* = 0.739	*p* = 1	*p* = 1
**Scholar grade**						
4th primary	7	6	7	χ^2^ = 1.55, *p* = 0.818		
5th primary	3	2	6			
6th primary	9	6	8			
**Gender**						
Male	11	10	13	χ^2^ = 0.65, *p* = 0.723		
Female	8	4	8			
**SES^a^**						
Low	0	6	2	χ^2^ = 15.71, *p* = 0.003		
Medium	15	8	17			
High	4	0	1			
Raven test (IQ)	99.05 (14.4)	99.7 (12.1)	102.2 (13.5)	*p* = 1	*p* = 0.665	*p* = 0.652
**CELF-4**						
Core language (percentile)	70.3 (14.1)	17.4 (4.7)	59.2 (14.4)	*p < 0.001	*p* = 0.119	*p < 0.001
**PROLEC-R**						
Written word decoding	114.3 (39.5)	86.8 (19.8)	67.7 (33.1)	*p* = 0.141	*p < 0.001	*p* = 0.138

*M, mean; SD, Standard deviation; IQ, Non-verbal Intelligence Quotient; Con, Control group; DLD, Developmental Language Disorder group; RD, Reading difficulties group*

*p < 0.05.

*^*a*^One missing data for SES in the RD group.*

With respect to the DLD group, all the children included in the remaining sample showed an objective significant language delay [percentile less than 25 in CELF-4 in the Clinical Evaluation of Language Fundamentals-4 Spanish Edition (Semel et al., 2003)], that had been persistent in time (all had a history of language difficulties, as informed by schools) generating a functional impact in their communication and learning (affecting, for example, their school learning). This impact in learning was also confirmed by their academic results. In this sense, children with DLD showed lower grade scores than their controls: DLD mean = 5.8 ± 0.59; Controls mean = 8.44 ± 0.55 (*p* < 0.0001); and higher retention rates than their control peers: DLD = 5; Controls = 0 (*p* < 0.0001).

On the other hand, all the children with RD of the sample showed reading decoding difficulties without language and cognitive difficulties with a language percentile higher than 25 on the CELF-4, a score of “Severe difficulty” or “Difficulty” on word decoding, as measured by the ratio between correctly read words and their reading time in the test *Batería de Evaluación de los Procesos Lectores revisada* (PROLEC-R. Battery of Evaluation of the Reading Processes, Revised) (Cuetos et al., 2013), and an IQ standard score greater than 75 in the Raven Matrix Test.

Finally, none of the children in the control group showed reading, oral language, or cognitive difficulties (schools informed of typical development and all tests presented values in normative ranges regarding IQ, oral language, and reading performance). After these confirmatory evaluations and questionnaires, six children were excluded from the sample because criteria were not met (*N* = 107).

For this study, we included children who accomplished all criteria for each group, who had data available at the same longitudinal moment for each of the three sources of evaluation (teacher, peers, and self-assessment). As a result, only 54 students (20 females; 37% of the sample) aged between 8 and 13 years could be included in the present study, mainly due to the low number of teacher reports and sociograms collected at the same moment. All participants were Spanish-Catalan bilinguals.

As mentioned, each child was classified into one of three different groups, resulting in a group of 14 children with DLD (4 females), a group of 21 children with RD (8 females), and a group of 19 Control participants (8 females). Groups were equivalent in terms of Age [*H*(2) = 0.471, *p* = 0.790], Non-verbal IQ [*H*(2) = 0.834, *p* = 0.659], grade and gender (see Table 1). As expected, oral language scores were lower for the DLD group [*H*(2) = 33.8, *p* < 0.001] and scores on written word decoding (the rate between the correct word reading divided by the total reading time) were lower for the RD group [*H*(2) = 14.3, *p* < 0.001] as compared to the other two groups. Socioeconomic status (SES) also differed between groups (χ^2^ = 15.71, *p* = 0.003), with lower SES in DLD children, an aspect that is common, according to recent reports (Bishop et al., 2016). To further explore the potential influence of SES, a MANCOVA was carried out with Group as a factor and SES as a covariate on all dependent variables of the present study, and results revealed that the covariate had no significant influence (absence of interactions between the covariate and the group for each dependent variable; *p*s > 0.087). The descriptive statistics of each group can be seen in Table 1.

### Materials

We used different instruments, which were answered by different agents (i.e., teacher, classmates, and students) to measure social status and peer relationships, social problems, adaptive skills, and bullying victimization.

To assess peer-rated prosocial behavior and victimization we used the sociometric test based on peer nomination *Conducta y Experiencias Sociales en Clase* (CESC: Behavior and social experiences in class) (Collell and Escudé, 2006), which is based on the model of Coie et al. (1982). Each student is individually asked to assign a series of roles among their classmates. This sociometric peer nomination uses items such as “Who does help others?” to evaluate prosocial behavior or “Who does get hit?” to evaluate victimization. This is the recommended sociogram by the *Institut per a la Convivència i l’Èxit escolar* [Institute for the coexistence and scholar success] of the Balearic Government for the prevention and early intervention in bullying situations. The CESC asks different questions to all the students in the class and records all peer nominations. Its internal consistency is between α = 0.82 and α = 0.88 for the current study.

Regarding adaptive skills, withdrawal and rejection as informed by teachers, we administered the Spanish teacher version of the Behavior Assessment System for Children (Reynolds and Kamphaus, 1992; BASC; González et al., 2004). This is a multidimensional evaluation instrument which assesses children’s and adolescents’ behavior and emotional status, including both clinical and adaptive variables. This version, which is responded by the schoolteachers, is composed of 149 items, which are answered using a four-level Likert scale (from “never” to “almost always”). In this study, we used three different dimensions of the BASC-T: social skills (measures social behaviors and adequacy, e.g., “He/she gives advice without offending others”), withdrawal (measures the tendency to avoid others and to isolation, sometimes, because of feeling rejected by peers, e.g., “He/she avoids other children”) and adaptability (measures flexibility to changes and ability to share things with others, e.g., “He/she has a good adaptation to routine changes”). The BASC internal consistency varies between α = 0.71 and α = 0.89 in our sample depending on the dimension evaluated.

Referring to self-reported social relationships and problems, we applied the Spanish self-reported version of the BASC (Reynolds and Kamphaus, 1992; González et al., 2004). As with the teacher version, the BASC-S assesses personality and behavior in children and adolescents themselves, from a clinical and an adaptive perspective. This version is composed of 146 items, and it is answered by means of a true or false response. In this study, we used two different dimensions of the instrument: social stress (which measures the level of anxiety experienced in social situations, e.g., “I feel that I only disturb other people”) and interpersonal relationships (which assesses the success and satisfaction experienced when socializing with others; e.g., “People like being with me”). For this version, internal consistency scores ranged between α = 0.79 and α = 0.80 in our sample depending on the dimension evaluated.

To measure bullying victimization assessed by children themselves, we used the Spanish version of the European Bullying Intervention Project Questionnaire (EBIP-Q; Brighi et al., 2012; Ortega-Ruiz et al., 2016). The EBIP-Q is composed by 14 items: seven of them measure self-perceived victimization, and the other half provide information about the participants’ self-perceived aggression. For the present study, we only considered victimization scores. The EBIP-Q assesses victimization yielding a total score with a cutoff point of 7, which allows for the classification of the respondent as a victim of severe bullying or not (González-Cabrera et al., 2018). Nevertheless, the ensemble of items is comprised of different forms of bullying behaviors, including verbal (e.g., “Someone has insulted me”), physical (e.g., “Someone has hit, kicked or pushed me”), instrumental (e.g., “Someone has stolen or broken my things”) and relational aspects (e.g., “I have been excluded, isolated or ignored by other people”). All items are answered using a five-level Likert scale which measures the frequency of the behaviors (from “never” to “more than once a week,” referring to the last 2 months). EBIP-Q internal consistency of the victimization scale was α = 0.71 for the current study.

### Procedure

The research ethics committee of the University of the Balearic Islands approved this study’s procedure and provided full consent. Moreover, all parents of students participating signed an informed written consent at the beginning of the study. Prior to the assessment, all children provided verbal agreement to participate in the sessions and they were informed that they could withdraw from the study at any moment.

All instruments were applied during the same scholar course. Both the EBIP-Q and the BASC-S were administered individually by our team members (properly trained psychologists and pedagogues) at the schools, accomplishing the optimal conditions for the tests. Meanwhile, the school centers administered the sociograms (CESC) to all class groups at mid-year. Finally, the teachers of all participants completed the teacher rating scale of the BASC. All responses were checked, processed and coded by our team following each test indications. CESC raw scores were transformed into Z-scores considering the mean and SD of each child’s class to make comparable different classrooms with an unequal number of students. The number of children ranged from 20 to 28 students per classroom (*M* = 24.93 ± 1.98; *Mdn* = 25). Raw scores were used for the other scales. Data were analyzed with SPSS version 25 and JASP version 0.14.0.0. No *p*-value correction for multiple comparisons was conducted for correlations. Since data did not accomplish the necessary parametric assumptions (lack of normal distribution, likely because of the reduced number of participants in each group), non-parametric analyses were performed.

## Results

### Results for Each Questionnaire

This section reports the results regarding the perception of teachers, students, and classroom peers separately by presenting the outcomes of each questionnaire independently of each other’s (see Table 2).

**TABLE 2:** Main outcomes of the questionnaires administered by group.

Type, Questionnaire and Scale	Control		DLD		RD		Kruskal-Wallis	
	*M*	SD	*M*	SD	*M*	SD	*H*	*p*
**Peer-report (CESC)**								
Prosociality	1	1.4	–0.1	0.7	–0.2	0.8	9.7	0.008
Victimization (general)	–0.5	0.4	0.6	1.3	–0.1	0.7	9.6	0.008
Physical victimization	–0.2	0.7	0.5	1.2	0.3	1	4.0	0.136
Verbal victimization	–0.3	0.5	0.5	1.3	–0.1	0.8	4.1	0.126
Relational victimization	–0.4	0.5	1	1.2	–0.3	0.6	12.2	0.002
**Teacher-report (BASC-T)**								
Social skills	23.8	5.6	15.2	5.8	22.3	6.8	12.6	0.002
Adaptability	16.2	2.5	11.9	3.3	14.7	3.6	10.9	0.004
Withdrawal	3.0	3.1	9.6	4.7	4.7	3.8	14.1	<0.0001
**Self-report**								
Social stress (BASC-S)	2.2	2.4	3.5	2.6	2.7	2.6	2.7	0.264
Interpersonal relations (BASC-S)	8.5	1. 2	7.7	2.2	7.8	1.8	3.2	0.2
Victimization (EBIP)	3.5	2.8	5.5	5.1	4.3	3.6	1.0	0.614

*DLD, developmental language disorder; RD, reading difficulties; CESC measures are expressed in Z-scores to make comparable different classrooms with different number of students; BASC and EBIP measures are expressed in raw scores.*

#### Perception of Classroom Peers: Conducta y Experiencias Sociales en Clase

With respect to the CESC results, the Kruskal-Wallis test performed showed differences between groups for the prosociality, *H*(2) = 9.721, *p* = 0.008, η_*H*_^2^ = 0.112, the relational victimization, *H*(2) = 12.221, *p* = 0.002, η_*H*_^2^ = 0.161, and the general victimization scores, *H*(2) = 9.571, *p* = 0.008, η_*H*_^2^ = 0.109. No other CESC variables reached significance (see Table 2).

**FIGURE 1:**
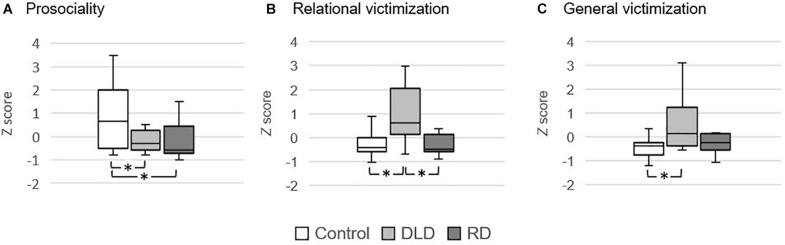
Group boxplots representing the scores on the CESC for the **(A)** prosociality, **(B)** relational victimization, and **(C)** general victimization scores. All measures are expressed in Z-scores (0, 1). ^∗^*p* < 0.05.

Follow-up Dunn *post hoc* tests revealed that the Control group was the most prosocial as compared to both the DLD (*p* = 0.042) and the RD group (*p* = 0.004). RD was as prosocial as the DLD group (*p* = 0.238; see Figure 1A).

While participants of the Control and the RD groups showed low and similar relational victimization scores (*p* = 0.392), the DLD group was more victimized than the other two groups (*p*s < 0.003; see Figure 1B). Regarding the general victimization score, the Control group obtained the lowest scores and did not differ from the RD group (*p* = 0.081). Moreover, participants in the DLD group did not significantly differ from those in the RD group (*p* = 0.081), yet they obtained higher general victimization scores than the Control group (*p* = 0.003; see Figure 1C).

#### Perception of Teachers: BASC-T

The Kruskal-Wallis test performed on the BASC scores responded by teachers showed differences between groups in social skills, *H*(2) = 12.558, *p* = 0.002, η_*H*_^2^ = 0.168, adaptability, *H*(2) = 10.939, *p* = 0.004, η_*H*_^2^ = 0.136, and withdrawal, *H*(2) = 14.08, *p* < 0.0001, η_*H*_^2^ = 0.198 (see Table 2).

**FIGURE 2:**
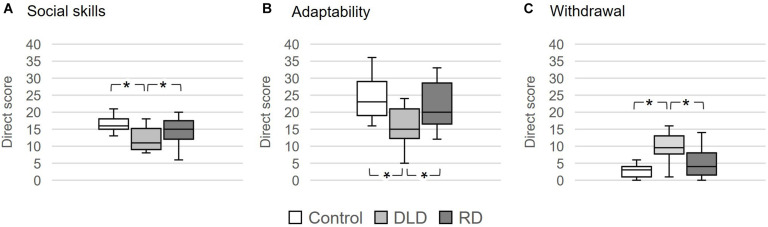
Group boxplots representing the scores on the BASC-T for **(A)** social skills, **(B)** adaptability, and **(C)** withdrawal scores. *p < 0.05.

Dunn *post hoc* tests showed that children with DLD were perceived as less socially skilled than both the Control and the RD groups (*p*s < 0.007), with no difference between the latter groups (*p* = 0.180; see Figure 2A). This pattern was paralleled for both the adaptability and withdrawal scores.

Regarding adaptability, both the Control and RD children were similar between each other (*p* = 0.106) and more skilled at adapting than the DLD group (*p*s < 0.027; see Figure 2B). Furthermore, the Control and RD groups were also equivalent (*p* = 0.108) and showed less withdrawal than children in the DLD group (*p*s < 0.008; see Figure 2C).

#### Self-Perception: BASC-S and EBIP-Q

The Kruskal-Wallis test performed on the BASC filled by the student showed that groups were similar in terms of their self-reported social stress and interpersonal relations (see Table 2). Also, the Kruskal-Wallis test performed on the EBIP-Q showed no differences between groups regarding the victimization score (see Table 2).

#### Associations Between Questionnaires

This section explores to what extent the outcomes of the scores of the different questionnaires administered in this study concur and can offer comparable results for both all participants and the different groups separately. Spearman correlation analyses were conducted to inspect the relation between the most relevant scores of all questionnaires for all participants (see Table 3).

**TABLE 3:** Spearman correlations between scores for all participants (*n* = 54).

		Peer-report		Teacher-report			Self-report			Language
		CESC		BASC-T			BASC-S		EBIP	CELF-4
Scale		1	2	3	4	5	6	7	8	9
1	Prosociality	1								
2	Victimization (general)	−0.28*	1							
3	Social skills	0.36**	−0.31*	1						
4	Adaptability	0.38***	−0.42***	0.62***	1					
5	Withdrawal	–0.19	0.17	−0.49***	−0.41***	1				
6	Social stress	0.12	0.22	0.02	0.04	0.11	1			
7	Interpersonal relations	0.07	–0.25	0.01	–0.23	0.06	−0.50***	1		
8	Victimization	–0.15	0.21	0.07	–0.18	–0.05	0.34**	−0.04	1	
9	Core language (Pc)	0.26	−0.37*	0.4*	0.38*	−0.42*	–0.25	0.22	−0.12	1

*Correlation coefficients (ρ) are displayed below the diagonal.*

Pc, percentile; *p < 0.05 (two-tailed); **p < 0.01 (two-tailed); ***p < 0.001 (two-tailed).

As shown in Table 3, all scores answered by the same agents were correlated with each other (except self-report measures of victimization and interpersonal relations). Moreover, both measures of the CESC questionnaire answered by classroom peers (prosociality and general victimization) showed associations with all scores of the BASC-T answered by teachers (social skills and adaptability), except for withdrawal (see Table 3). These correlations followed the expected directions (note that for both general victimization and withdrawal, higher scores reflect undesired outcomes). However, neither the CESC (reported by peers) nor the BASC-T (reported by teachers) scores correlated with the BASC-S or the EBIP self-report measures. Furthermore, the social stress score of the BASC-S and the victimization measure of the EBIP correlated positively, showing that the association of these two negative characteristics is coherent. In the same vein, the objective measure of language level (percentile in the core language score, as measured by the CELF-4) showed significant correlations with most of the peer and teacher reports, but not with self-reports. Higher language scores were associated with lower general victimization and withdrawal, as measured by peers and tutors, respectively. In contrast, both social skills and adaptability showed significant positive correlations with higher scores in the CELF-4.

Moreover, the Fisher’s *Z* conducted to examine whether the correlation pattern differed among the Control, the DLD and the RD groups in the most relevant and relatable measures between questionnaires yielded non-significant results for each group comparison (see Table 4). These results provide evidence on the similarity of associations among groups for the most related measures between different questionnaires.

**TABLE 4:** Spearman correlation coefficients (ρ) of comparable scores belonging to different questionnaires for each group separately (*n*_Control_ = 19; *n*_DLD_ = 14; *n*_RD_ = 21) and Fisher’s *Z*.

		Fisher’s *Z*								
	
		ρ			Con-DLD		Con-RD		DLD-RD	
Score 1	Score 2	Con	DLD	RD	*Z*	*p*	*Z*	*p*	*Z*	*p*
Social skills (BASC-T)	Prosociality (CESC)	0.361**	0.440^+^	0.533*	–0.24	0.810	–0.63	0.529	–0.32	0.750
Social skills (BASC-T)	Int relations (BASC-S)	0.011	–0.189	–0.196	0.52	0.606	0.61	0.542	0.02	0.985
Prosociality (CESC)	Int relations (BASC-S)	0.070	0.037	–0.07	0.08	0.933	0.41	0.683	0.28	0.780
Gral. victimization (CESC)	Victimization (EBIP)	0.206	–0.057	0.046	0.68	0.497	0.47	0.635	–0.27	0.788

*Con, Control group; DLD, Developmental language disorder group; RD, Reading difficulties group; Int. relations, Interpersonal relations; Gral. Victimization, victimization (general).*

*p < 0.05 (two-tailed); **p < 0.01 (two-tailed); ^+^p < 0.06 (two-tailed).

## Discussion

The current study aims to analyze bullying experiences and related variables in primary school children with DLD and RD compared to their age-matched peers using teacher reports, peer reports, and self-reports on victimization. Previous research has demonstrated that students with DLD and RD have a higher prevalence of peer problems, poorer acceptance of their classmates, lower quality and quantity of friendships, and larger vulnerability to peer rejection (Laws et al., 2012; Mok et al., 2014; Lloyd-Esenkaya et al., 2020). However, the accumulating evidence indicates the necessity to distinguish between the perspectives of different reporters and consider the students’ characteristics (Sidera et al., 2020; Gough Kenyon et al., 2021).

Our results indicate that children with DLD and RD differ from their typically developing peers in social and emotional skills when these are reported by others. In line with other studies (Andrés-Roqueta et al., 2016; Sako, 2016), we find lower levels of prosocial skills in DLD and RD students compared to control students in peer measures, reflecting the significant difficulties these children can have with social integration and acceptance in the group. A deficit of prosocial behavior could indicate a poor emotional understanding and lack of learning in helping behavior offered to others (Lloyd-Esenkaya et al., 2020), but also could reflect their insufficient language level to hold up prosocial behaviors (Fujiki et al., 2013; Andrés-Roqueta et al., 2016; Sako, 2016).

Furthermore, in the line of previous investigations of peer victimization (Andrés-Roqueta et al., 2016), our work points toward a higher level of victimization as assessed by peers, but only for students with DLD. These results appear to align with the teachers’ ratings confirmed in our study and previous ones (Conti-Ramsden and Botting, 2004; Bakopoulou and Dockrell, 2016). Moreover, the teachers’ rating indicates that students with DLD present poor social skills, less adaptability, and more social interaction withdrawal. These results show that, in the views of both peers and teachers, students with DLD (but not those with RD) have less social competence than their peers. This social deficit, in conjunction with their language difficulties, may modulate their higher tendency to peer victimization.

This finding suggests that the DLD and RD groups present different profiles in terms of their socio-emotional skills, and not only regarding the nature and severity of their language difficulties, which are more severe in children with DLD (Laws et al., 2012; Mok et al., 2014; Lloyd-Esenkaya et al., 2020). However, we cannot establish whether there is a direct relationship between language skills and peer difficulties (Van den Bedem et al., 2018b). Although previous studies have failed to predict social profiles from language level, we have found a significant correlation between a lower language level and both a larger peer-rated victimization and teacher-rated larger withdrawal and poorer social skills, as rated by peers and teachers, respectively (Lindsay and Dockrell, 2012). It is probable that difficulties in emotional and social competence among children with DLD comprise an additional risk factor for victimization added to their communication problems. In line with this possibility, van der Wilt et al. (2018) state that communicative skills and social problems are related in a cyclic way: language difficulties make children less eager to socially interact with others, having less opportunities of training and developing their communicative skills, thus likely making them more prone to peer rejection. Future studies should further explore the relationship between these variables.

In summary, our results related to the perception of others (teachers and peers) show less prosocial behaviors in both groups with language difficulties, but less social skills, less adaptability, more withdrawal behaviors, and more victimization only in the group with DLD compared to the other two groups and reported by other informants. Nevertheless, self-reports informed of equal social relations, social stress, and peer victimization between groups. Altogether, this seems to reveal that the perception of others (regardless of whether it was the teacher or the peers of the participants) tends to be similar, and not associated with the self-perceived situation, as confirmed by correlations between measures. In this sense, our results show that the teacher and peer reports judge DLD individuals as less socially skilled and more victimized, while students with DLD have a more positive and perhaps biased perception of their social skills and their peer relationships (Undheim et al., 2011; Wadman et al., 2011).

One explanation, in line with the model of social information processing (Crick and Dodge, 1994), would indicate that children develop the perception and the interpretation of context cues differently from other agents involved in the same situation. In our case, the students with DLD could be wrongly understanding and interpreting the social situation, showing a lack of prosociality. Deficits in prosociality and language skills can increase the risk of being victimized, as bullies could perceive children with DLD as less integrated or even awkward, justifying their relational victimization toward them. Hence, the wrong understanding and comprehension of the situation that DLD children have could explain that they do not perceive themselves as less prosocial, with less social skills and as victims.

Victimized students may not acknowledge the experience of rejection or victimization, as seems to happen to the children with DLD in our sample (who are rated as more victimized by others, but not by themselves). In this vein, some studies have found that only a low percentage of adolescents (between 16 and 32%) who are categorized as bullying victims perceive themselves as victims (Hwang et al., 2017; Sidera et al., 2020). Sidera et al. (2020) explain that this misperception can be related to the type and quantity of bullying actions that someone experiences. These students may not be aware of being disliked if they are not overtly disliked and treated aversively by their peers (García Bacete et al., 2018). Thus, some victimized students may habituate to direct verbal and physical aggression and would normalize such behaviors. However, although these children do not label these behaviors as victimization, they suffer their emotional consequences (Valera-Pozo et al., 2020). In this sense, authors such as Kilpatrick et al. (2019) found that bullying mediated the effect between a history of DLD and internalizing symptomatology (depression, anxiety, etc.). This situation might lead to a more obvious sociometric profile of rejection by class peers (García Bacete et al., 2018).

These arguments may explicate the differences between teacher reports, the perceptions of classmates, and children’s self-reports. Other studies have also found that peer reports might be more reliable that self-reports for the assessment of bullying. For example, Schuster (1999) evaluated a large sample of students in upper secondary school and concluded that peer reports might be more reliable than self-reports when assessing bullying behaviors. Moreover, similar to our data, peer-reports were more in line with teacher’s reports than with self-reports (Schuster, 1999). More recently, Košir et al. (2020) revealed, in a sample of elementary school students (early adolescents), that predictors of victimization are different when evaluated through peer-reports, compared with self-reports. In view of this, future studies might want to explore more in depth the reliability and validity of self, peer, and teacher reports of bullying in children with DLD and RD.

Finally, there is another interesting finding in our study. As abovementioned, the DLD group presented lower SES than the other two groups, although this difference did not affect any of the dependent variables measured. Even if this result is surprising, it is not uncommon than socioeconomic variables could be related to the presence of DLD in children and youngsters. Thus, some recent studies have showed that low familiar socioeconomic status (SES) can be associated with decreases in the quality and the quantity of the linguistic input received by children, besides their lexical skills and their possibilities to access assessments and treatments (Hirsh-Pasek et al., 2015; Gilkerson et al., 2017; Romeo et al., 2018; Auza-Benavides et al., 2019; Sureda-García et al., 2019). These deficits are often related with a poorer language development, which can be compatible with DLD (Ferinu et al., 2021). Moreover, DLD has a considerable genetic component, and some contextual factors might be in part a result of genetics. For instance, families of children with DLD usually have a history of language impairment that can lead to a lower level of studies, lower incomes and, consequently, a lower familiar SES (Conti-Ramsden et al., 2018; Hart et al., 2021).

Our study has different psychological and educational implications. The first of them is the need to make teachers aware that, although the relation between language problems and peer rejection is complicated and might be mediated by other variables, students with DLD are at high risk of showing social skills difficulties and being victimized. The second refers to the profit of introducing more socioemotional programs such as the training of prosocial behaviors, social skills, and emotional awareness, especially in the case of students with DLD and RD (Lloyd-Esenkaya et al., 2020). The third implication is the importance of providing social support to teachers and families, to improve their understanding of how children perceive victimization. By doing so, these agents could teach students the exact situations where bullying appears. In turn, this additional support could help reduce the widespread tolerance and normalization of bullying behaviors. Finally, it would be relevant to promote close friendship as an important protective factor against risk for the students with DLD and RD, which could be useful to decrease their levels of victimization (Redmond, 2011). Several studies show that training certain skills in children and adolescents, such as conflict resolution, anger management, the ability to forgive others, and even general social skills, can be useful to help to develop friendships and improve closeness and supportiveness (Bollmer et al., 2005; Estell et al., 2009; Barcaccia et al., 2018). More specifically, despite there are not many interventions in this regard, the Fast Friend program has demonstrated to be a good option for promoting close friendships in the school (Echols and Ivanich, 2021). This method consists of a variable number of sessions in which students are encouraged to work in pairs with peers. In the first sessions, they ask and answer questions that gradually become more and more personal. In the final session, children are proposed to solve a problem together.

Despite these valuable results, our study must be interpreted cautiously due to some limitations, being the main of them the small sample size, which complicates the generalization of the findings. It would be desirable for further studies to also include older children. Although the age range of children included in the present study is critical for studying the bullying phenomenon, it would be advisable to explore it during compulsory secondary education (ages 12–15 approximately). Moreover, since this study investigates the contrast between bullying self-perception with peer and teacher perceptions, it is advisable to directly ask the participants about their own perceptions of being bullied or not through open questions. More in detail, reasons why children with DLD rate themselves as less victimized than peers do are of great interest for both clinical and educational purposes. Future works might want to elucidate whether children with DLD have less consciousness about being bullied, poor emotional understanding, and/or experience more feelings of shame and guilt than their normative peers. Finally, further investigation in this field should address other aspects, such as gender, physical appearance, personal hygiene, behavioral adjustment, feelings of shame and guilt and sociocultural variables, which can also intervene in the victimization phenomenon.

In sum, children with DLD and RD show less prosocial behaviors reported by peers. Besides, children with DLD show fewer social skills, less adaptative behaviors, more withdrawal conducts and suffer more victimization, as reported by peers and teachers. Nevertheless, self-reports do not evidence any difference between groups. These results reveal an incongruence between self-reports and other-informant-reports and should be studied in future research. Despite this incongruence, social and emotional aspects of children with DLD or RD should be attended to provide the skills they need to confront and cope with bullying and similar situations.

## Data Availability Statement

The raw data supporting the conclusions of this article will be made available by the authors under request, without undue reservation.

## Ethics Statement

The studies involving human participants were reviewed and approved by the University of the Balearic Islands. The patients/participants provided their written informed consent to participate in this study.

## Author Contributions

IS-G designed the study, analyzed the data, and wrote the first draft of the manuscript. MV-P designed the study, collected and curated the data, analyzed the data, wrote, and corrected the manuscript. VS-A curated the data, analyzed the data, wrote, and corrected the manuscript. DA-R designed the study, wrote, and corrected the manuscript. EA-M designed the study, analyzed the data, wrote, and corrected the manuscript. All authors contributed to the article and approved the submitted version.

## Conflict of Interest

The authors declare that the research was conducted in the absence of any commercial or financial relationships that could be construed as a potential conflict of interest.

## Publisher’s Note

All claims expressed in this article are solely those of the authors and do not necessarily represent those of their affiliated organizations, or those of the publisher, the editors and the reviewers. Any product that may be evaluated in this article, or claim that may be made by its manufacturer, is not guaranteed or endorsed by the publisher.

## References

[B1] Adriaensens S. Van Waes S. Struyf E. (2017). Comparing acceptance and rejection in the classroom interaction of students who stutter and their peers: a social network analysis. *J. Fluency Disord.* 52 13–24. 10.1016/j.jfludis.2017.02.002 28576290

[B2] Andrés-Roqueta C. Adrian J. E. Clemente R. A. Villanueva L. (2016). Social cognition makes an independent contribution to peer relations in children with specific language impairment. *Res. Dev. Disabil.* 49-50 277–290. 10.1016/j.ridd.2015.12.015 26745788

[B3] Auza-Benavides A. Peñaloza C. Murata C. (2019). “The influence of maternal education on the linguistic abilities of monolingual Spanish-speaking children with and without specific language impairment’,” in *Atypical Language Development in Romance Languages*, eds Aguilar-Mediavilla E. Buil-Legaz L. López-Penadés R. Sanchez-Azanza V. A. Adrover-Roig D. (Amsterdam: John Benjamins Publishing Company), 93–112. 10.1075/z.223.06ale

[B4] Bakopoulou I. Dockrell J. E. (2016). The role of social cognition and prosocial behaviour in relation to the socio-emotional functioning of primary aged children with specific language impairment. *Res. Dev. Disabil.* 4 354–370. 10.1016/j.ridd.2015.12.013 26773217

[B5] Barcaccia B. Pallini S. Baiocco R. Salvati M. Saliani A. M. Schneider B. H. (2018). Forgiveness and friendship protect adolescent victims of bullying from emotional maladjustment. *Psicothema* 30 427–433. 10.7334/psicothema2018.11 30353845

[B6] Berger C. Rodkin P. C. (2009). Male and female victims of male bullies: Social status differences by gender and informant source. *Sex Roles* 61 72–84. 10.1007/s11199-009-9605-9

[B7] Bhakta P. Hackett R. J. Hackett L. (2002). The prevalence and associations of reading difficulties in a population of South Indian children. *J. Res. Read.* 25 191–202. 10.1111/1467-9817.00168

[B8] Bishop D. V. M. Snowling M. J. (2004). Developmental dyslexia and specific language impairment: same or different? *Psychol. Bull.* 130:858. 10.1037/0033-2909.130.6.858 15535741

[B9] Bishop D. V. M. Snowling M. J. Thompson P. A. Greenhalgh T. (2017). Phase 2 of CATALISE: a multinational and multidisciplinary Delphi consensus study of problems with language development: terminology. *J. Child Psychol. Psychiatry Allied Discip.* 58 1068–1080. 10.1111/jcpp.12721 28369935 PMC5638113

[B10] Bishop D. V. M. Snowling M. J. Thompson P. A. Greenhalgh T. Catalise Consortium. (2016). CATALISE: a multinational and multidisciplinary Delphi consensus study. identifying language impairments in children. *PLoS One* 11:1–26. 10.1371/journal.pone.0158753 27392128 PMC4938414

[B11] Bollmer J. Milich R. Harrison J. Maras M. (2005). A friend in need: the role of friendship quality as a protective factor in peer victimization and bullying. *J. Interpers. Violence* 20 701–712. 10.1177/0886260504272897 15851537

[B12] Bouman T. van der Meulen M. Goossens F. A. Olthof T. Vermande M. M. Aleva E. A. (2012). Peer and self-reports of victimization and bullying: their differential association with internalizing problems and social adjustment. *J. Sch. Psychol.* 50 759–774. 10.1016/j.jsp.2012.08.004 23245499

[B13] Boyer M. E. Leitão S. Claessen M. Badcock N. A. Nayton M. (2019). Correlates of externalising and internalising problems in children with dyslexia: an analysis of data from clinical casefiles. *Aust. Psychol.* 55 62–72. 10.1111/ap.12409

[B14] Brighi A. Ortega R. Pyzalski J. Scheithauer H. Smith P. K. Tsormpatzoudis C. (2012). *European Bullying Intervention Project Questionnaire (EBIPQ).* Bologna: University of Bologna.

[B15] Brinton B. Fujiki M. Montague E. C. Hanton J. L. (2000). Children with language impairment in cooperative work groups: a pilot study. *Lang. Speech. Hear. Serv. Sch.* 31 252–264. 10.1044/0161-1461.3103.252 27764443

[B16] Buil-Legaz L. Aguilar-Mediavilla E. Rodríguez-Ferreiro J. (2015). Reading skills in young adolescents with a history of specific language impairment: the role of early semantic capacity. *J. Commun. Disord.* 58 14–20. 10.1016/j.jcomdis.2015.08.001 26313625

[B17] Carrillo M. S. Alegría J. Miranda P. Sánchez Pérez N. (2011). Evaluación de la dislexia en la escuela primaria: prevalencia en español [assessment of dyslexia in primary school: prevalence in Spanish]. *Escritos Psicol. Psychol. Writ.* 4 35–44. 10.5231/psy.writ.2011.1407

[B18] Cecilia M. Vittorini P. Cofini V. di Orio F. (2014). The prevalence of reading difficulties among children in scholar age. *Styles Commun.* 6 18–30.

[B19] Cheng H. Furnham A. (2020). Correlates of maternal emotional stability: findings from the millennium cohort study. *Pers. Individ. Dif.* 164:110119. 10.1016/j.paid.2020.110119

[B20] Coie J. D. Dodge K. A. Coppotelli H. (1982). Dimensions and types of social status: a cross-age perspective. *Dev. Psychol.* 18 557–570. 10.1037/0012-1649.18.4.557

[B21] Collell C. J. Escudé C. (2006). Maltrato entre alumnos [I]. presentación de un cuestionario para evaluar les relaciones entre iguales. CESC conducta y experiencies sociales en clase [abuse between students [I]. questionnaire to evaluate equal relations. CESC: behavior and social experiences in class]. *Ámbits Psicopedag* 18 8–12.

[B22] Conti-Ramsden G. Botting N. (2004). Social difficulties and victimization in children with SLI at 11 years of age. *J. Speech, Lang. Hear. Res.* 47 145–161. 10.1044/1092-4388(2004/013) 15072535

[B23] Conti-Ramsden G. Botting N. (2014). “Educational placements for children with specific language impairments,” in *Speech and Language Impairments in Children: Causes, Characteristics, Intervention and Outcome*, eds Bishop D. V. M. Leonard L. (Hove: Psychology Press).

[B24] Conti-Ramsden G. Durkin K. Toseeb U. Botting N. Pickles A. (2018). Education and employment outcomes of young adults with a history of developmental language disorder. *Int. J. Lang. Commun. Disord.* 53 237–255. 10.1111/1460-6984.12338 29139196 PMC5873379

[B25] Cook C. R. Williams K. R. Guerra N. G. Kim T. E. (2010). “Variability in the prevalence of bullying and victimization: a cross-national and methodological analysis,” in *Handbook of Bullying in Schools: An International Perspective*, eds Jimerson S. R. Swearer S. M. Espelage D. L. (New York, NY: Routledge), 347–362.

[B26] Crick N. R. Dodge K. A. (1994). A review and reformulation of social information-processing mechanisms in children’s social adjustment. *Psychol. Bull.* 115 74–101. 10.1037/0033-2909.115.1.74

[B27] Cuetos F. Rodríguez B. Ruano E. Arribas D. (2013). *PROLEC-R. Bateria de Evaluación de los Procesos Lectores, Revisada [PROLEC-R. Battery of Evaluation of the Reading Processes, Revised].* Madrid: Ediciones, TEA.

[B28] Echols L. Ivanich J. (2021). From “fast friends” to true friends: can a contact intervention promote friendships in middle school? *J. Res. Adolesc.* 16 1–20. 10.1111/jora.12622 33998093

[B29] Ellenbogen M. A. Hodgins S. (2004). The impact of high neuroticism in parents on children’s psychosocial functioning in a population at high risk for major affective disorder: a family-environmental pathway of intergenerational risk. *Dev. Psychopathol.* 16 113–136. 10.1017/S0954579404044438 15115067

[B30] Erozkan A. (2013). The effect of communication skills and interpersonal problem solving skills on social self-efficacy. *Educ. Sci. Theory Pract.* 13 739–745.

[B31] Estell D. Jones M. Pearl R. Acker R. (2009). Best friendships of students with and without learning disabilities across late elementary school. *Except. Child.* 76 110–124. 10.1177/001440290907600106

[B32] Ferinu L. Ahufinger N. Pacheco-Vera F. Sanz-Torrent M. Andreu L. (2021). Antecedentes familiares, factores sociodemográficos y dificultades lingüísticas en el trastorno del desarrollo del lenguaje [family history, sociodemographic factors and language difficulties in language development disorder]. *Rev. Logop. Foniatría y Audiol.* 41 29–39. 10.1016/j.rlfa.2020.01.003

[B33] Font-Jordà A. Gamundí A. Nicolau Llobera M. C. Aguilar-Mediavilla E. (2018). Use of the 2D:4D digit ratio as a biological marker of specific language disorders. *An. Pediatría* 89 361–368. 10.1016/j.anpede.2018.02.008 29625804

[B34] Forrest C. L. Gibson J. L. St Clair M. C. (2021). Social functioning as a mediator between developmental language disorder (DLD) and emotional problems in adolescents. *Int. J. Environ. Res. Public Health* 18:1221. 10.3390/ijerph18031221 33572993 PMC7908163

[B35] Fujiki M. Brinton B. Isaacson T. Summers C. (2001). Social behaviors of children with language impairment on the playground. *Lang. Speech. Hear. Serv. Sch.* 32 101–113. 10.1044/0161-1461(2001/008) 27764354

[B36] Fujiki M. Brinton B. McCleave C. P. Anderson V. W. Chamberlain J. P. (2013). A social communication intervention to increase validating comments by children with language impairment. *Lang. Speech. Hear. Serv. Sch.* 44 3–19. 10.1044/0161-1461(2012/11-103) 23305940

[B37] García Bacete F. J. Sureda-García I. Muñoz-Tinoco V. Jiménez-Lagares I. Marande Perrin G. Rosel J. F. (2018). Interpersonal perceptions of adverse peer experiences in first-grade students. *Front. Psychol.* 9:1165. 10.3389/fpsyg.2018.01165 30042712 PMC6048420

[B38] Ghisi M. Bottesi G. Re A. M. Cerea S. Mammarella I. C. (2016). Socioemotional features and resilience in Italian university students with and without dyslexia. *Front. Psychol.* 7:1–9. 10.3389/fpsyg.2016.00478 27065220 PMC4814487

[B39] Gilkerson J. Richards J. A. Warren S. F. Montgomery J. K. Greenwood C. R. Kimbrough Oller D. (2017). Mapping the early language environment using all-day recordings and automated analysis. *Am. J. Speech-Language Pathol.* 26 248–265. 10.1044/2016_AJSLP-15-0169 PMC6195063 28418456

[B40] González J. Fernández S. Pérez E. Santamaría P. (2004). *Adaptación española del Sistema de Evaluación de la Conducta en Niños y Adolescentes: BASC [Spanish Adaptation of the Behavior Assessment System for Children and Adolescents: BASC].* Madrid: TEA Ediciones.

[B41] González-Cabrera J. Tourón J. León-Mejía A. Machimbarrena J. M. (2018). *Informe Ejecutivo del Proyecto CIBERAACC – Acoso y Ciberacoso en Estudiantes de Altas Capacidades: Prevalencia y Afectación Psicológica [Executive Report of the CIBERAACC Project – Harassment and Cyberbullying in Gifted Students: Prevalence and Psychological Affectation].* Logroño. Available online at: http://www.infocoponline.es/pdf/informeciberaacc.pdf (accessed March, 2018).

[B42] Gough Kenyon S. M. Palikara O. Lucas R. M. (2021). Consistency of parental and self-reported adolescent wellbeing: evidence from developmental language disorder. *Front. Psychol.* 12:629577. 10.3389/fpsyg.2021.629577 33776852 PMC7991577

[B43] Goulandris N. K. Snowling M. J. Walker I. (2000). Is dyslexia a form of specific language impairment? A comparison of dyslexic and language impaired children as adolescents. *Ann. Dyslexia* 50 103–120. 10.1007/s11881-000-0019-1 20563782

[B44] Graham S. Juvonen J. (1998). Self-blame and peer victimization in middle school: an attributional analysis. *Dev. Psychol.* 34 587–599. 10.1037/0012-1649.34.3.587 9597367

[B45] Hankin B. L. Abramson L. Y. (2001). Development of gender differences in depression: an elaborated cognitive vulnerability–transactional stress theory. *Psychol. Bull.* 127 773–796. 10.1037/0033-2909.127.6.773 11726071

[B46] Hart S. A. Little C. van Bergen E. (2021). Nurture might be nature: cautionary tales and proposed solutions. npj Sci. *Learn.* 6:2. 10.1038/s41539-020-00079-z 33420086 PMC7794571

[B47] Hawker D. S. J. Boulton M. J. (2000). Twenty years’ research on peer victimization and psychosocial maladjustment: a meta-analytic review of cross-sectional studies. *J. Child Psychol. Psychiatry* 41 441–455. 10.1111/1469-7610.00629 10836674

[B48] Hirsh-Pasek K. Adamson L. B. Bakeman R. Owen M. T. Golinkoff R. M. Pace A. (2015). The contribution of early communication quality to low-income children’s language success. *Psychol. Sci.* 26 1071–1083. 10.1177/0956797615581493 26048887

[B49] Hoover W. A. Gough P. B. (1990). The simple view of reading. *Read. Writ.* 2 127–160. 10.1007/BF00401799

[B50] Humphrey N. Mullins P. M. (2004). Self-concept and self-esteem in developmental dyslexia. *J. Res. Spec. Educ. Needs* 2 1–13. 10.1111/j.1471-3802.2002.00163.x

[B51] Hwang S. Shin Kim Y. Koh Y.-J. Bishop S. Leventhal B. (2017). Discrepancy in perception of bullying experiences and later internalizing and externalizing behavior: a prospective study. *Aggress. Behav.* 43 493–502. 10.1002/ab.21707 28326572

[B52] Ibáñez-Rodríguez A. Ahufinger N. Ferinu L. García-Arch J. Andreu L. Sanz-Torrent M. (2021). Dificultades sociales, emocionales y victimización específica por el lenguaje en el trastorno del desarrollo del lenguaje [social and emotional difficulties and specific victimization by language in language development disorder]. *Rev. Logop. Foniatría y Audiol.* 41 40–48. 10.1016/j.rlfa.2020.03.017

[B53] Kilpatrick T. Leitão S. Boyes M. (2019). Mental health in adolescents with a history of developmental language disorder: the moderating effect of bullying victimisation. *Autism Dev. Lang. Impair.* 4:239694151989331. 10.1177/2396941519893313

[B54] Knox E. Conti-Ramsden G. (2003). Bullying risks of 11-year-old children with specific language impairment (SLI): does school placement matter? *Int. J. Lang. Commun. Disord.* 38 1–12. 10.1080/13682820304817 12569033

[B55] Knox E. Conti-Ramsden G. (2007). Bullying in young people with a history of specific language impairment (SLI). *Educ. Child Psychol.* 24 130–141.

[B56] Koenig J. L. Barry R. A. Kochanska G. (2010). Reading difficult children: parents’ personality and children’s proneness to anger as predictors of future parenting. *Parent. Sci. Pract.* 10 258–273. 10.1080/15295192.2010.492038 21243035 PMC3018753

[B57] Košir K. Klasinc L. Špes T. Pivec T. Cankar G. Horvat M. (2020). Predictors of self-reported and peer-reported victimization and bullying behavior in early adolescents: the role of school, classroom, and individual factors. *Eur. J. Psychol. Educ.* 35 381–402. 10.1007/s10212-019-00430-y

[B58] Ladd G. W. Kochenderfer-Ladd B. (2002). Identifying victims of peer aggression from early to middle childhood: analysis of cross-informant data for concordance, estimation of relational adjustment, prevalence of victimization, and characteristics of identified victims. *Psychol. Assess.* 14 74–96. 10.1037/1040-3590.14.1.74 11911051

[B59] Laws G. Bates G. Feuerstein M. Mason-Apps E. White C. (2012). Peer acceptance of children with language and communication impairments in a mainstream primary school: associations with type of language difficulty, problem behaviours and a change in placement organization. *Child Lang. Teach. Ther.* 28 73–86. 10.1177/0265659011419234

[B60] Levickis P. Sciberras E. McKean C. Conway L. Pezic A. Mensah F. K. (2018). Language and social-emotional and behavioural wellbeing from 4 to 7 years: a community-based study. *Eur. Child Adolesc. Psychiatry* 27 849–859. 10.1007/s00787-017-1079-7 29143155 PMC6013518

[B61] Lindsay G. Dockrell J. E. (2012). Longitudinal patterns of behavioral, emotional, and social difficulties and self-concepts in adolescents with a history of specific language impairment. *Lang. Speech. Hear. Serv. Sch.* 43 445–460. 10.1044/0161-1461(2012/11-0069) 22826367

[B62] Lloyd-Esenkaya V. Forrest C. L. Jordan A. Russell A. J. Clair M. C. S. (2021). What is the nature of peer interactions in children with language disorders? A qualitative study of parent and practitioner views. *Autism Dev. Lang. Impair.* 6:239694152110053. 10.1177/23969415211005307 PMC9620689 36381529

[B63] Lloyd-Esenkaya V. Russell A. J. St Clair M. C. (2020). What are the peer interaction strengths and difficulties in children with developmental language disorder? A systematic review. *Int. J. Environ. Res. Public Health* 17:3140. 10.3390/ijerph17093140 32365958 PMC7246450

[B64] Lönnqvist J.-E. Verkasalo M. Mäkinen S. Henriksson M. (2009). High neuroticism at age 20 predicts history of mental disorders and low self-esteem at age 35. *J. Clin. Psychol.* 65 781–790. 10.1002/jclp.20571 19267331

[B65] Mok P. L. H. Pickles A. Durkin K. Conti-Ramsden G. (2014). Longitudinal trajectories of peer relations in children with specific language impairment. *J. Child Psychol. Psychiatry Allied Discip.* 55 516–527. 10.1111/jcpp.12190 24410167 PMC4283728

[B66] Monjas M. I. Martín-Antón L. J. García-Bacete F.-J. Sanchiz M. L. (2014). Rejection and victimization of students with special educational needs in first grade of primary education. *An. Psicol.* 30 499–511. 10.6018/analesps.30.2.158211

[B67] Olweus D. (2013). School bullying: development and some important challenges. *Annu. Rev. Clin. Psychol.* 9 751–780. 10.1146/annurev-clinpsy-050212-185516 23297789

[B68] Ortega-Ruiz R. Del Rey R. Casas J. A. (2016). Evaluar el bullying y el cyberbullying validación española del EBIP-Q y del ECIP-Q [assess bullying and cyberbullying Spanish validation of the EBIP-Q and the ECIP-Q]. *Psicol. Educ.* 22 71–79. 10.1016/j.pse.2016.01.004

[B69] Pipaş M. D. Jaradat M. (2010). Assertive communication skills. *Ann. Univ. Apulensis Ser. Oeconomica* 2 649–656. 10.29302/oeconomica.2010.12.2.17

[B70] Ramus F. Marshall C. R. Rosen S. Van Der Lely H. K. J. (2013). Phonological deficits in specific language impairment and developmental dyslexia: towards a multidimensional model. *Brain* 136 630–645. 10.1093/brain/aws356 23413264 PMC3572935

[B71] Raven J. C. Court J. H. Raven J. C. (1995). *Test de Raven - Matrices Progresivas [Raven’s Progressive Matrices Test].* Mexico: Pearson.

[B72] Redmond S. M. (2011). Peer victimization among students with specific language impairment, attention-deficit/hyperactivity disorder, and typical development. *Lang. Speech. Hear. Serv. Sch.* 42 520–535. 10.1044/0161-1461(2011/10-0078) 21844400 PMC4496414

[B73] Reynolds C. R. Kamphaus C. W. (1992). *Behavior Assessment System for Children (BASC).* Circle Pine, MN: American Guidance Services.

[B74] Romeo R. R. Leonard J. A. Robinson S. T. West M. R. Mackey A. P. Rowe M. L. (2018). Beyond the 30-million-word gap: children’s conversational exposure is associated with language-related brain function. *Psychol. Sci.* 29 700–710. 10.1177/0956797617742725 29442613 PMC5945324

[B75] Sako E. (2016). The emotional and social effects of dyslexia. *Eur. J. Interdiscip. Stud.* 4:233. 10.26417/ejis.v4i2.p233-241

[B76] Schmitz N. Kugler J. Rollnik J. (2003). On the relation between neuroticism, self-esteem, and depression: results from the National Comorbidity Survey. *Compr. Psychiatry* 44 169–176. 10.1016/S0010-440X(03)00008-7 12764703

[B77] Schuster B. (1999). Outsiders at school: the prevalence of bullying and its relation with social status. *Gr. Process. Intergr. Relations* 2 175–190. 10.1177/1368430299022005

[B78] Semel E. Wiig E. H. Secord W. A. (2003). *Clinical Evaluation of Language Fundamentals^®^*, 4th Edn. San Antonio, TX: Pearson, Psychological Corporation.

[B79] Sidera F. Serrat E. Collell J. Perpiñà G. Ortiz R. Rostan C. (2020). Bullying in primary school children: the relationship between victimization and perception of being a victim. *Int. J. Environ. Res. Public Health* 17 1–17. 10.3390/ijerph17249540 33419249 PMC7766212

[B80] Smith K. A. Barstead M. G. Rubin K. H. (2017). Neuroticism and conscientiousness as moderators of the relation between social withdrawal and internalizing problems in adolescence. *J. Youth Adolesc.* 46 772–786. 10.1007/s10964-016-0594-z 27844459 PMC5346328

[B81] Smith-Spark J. H. Henry L. A. Messer D. J. Ziêcik A. P. (2017). Verbal and non-verbal fluency in adults with developmental dyslexia: phonological processing or executive control problems? *Dyslexia* 23 234–250. 10.1002/dys.1558 28493359

[B82] Sureda-García I. Valera-Pozo M. Aguilar-Mediavilla E. (2019). “Afectación del lenguaje debido a variables sociales y emocionales [language difficulties due to social and emotional variables],” in *Dificultades del lenguaje en los trastornos del desarrollo, Vol. III: Factores de riesgo y dificultades comórbidas*, eds Aguilar-Mediavilla E. Igualada A. (Barcelona: Editorial UOC), 71–124.

[B83] Turunen T. Poskiparta E. Salmivalli C. (2017). Are reading difficulties associated with bullying involvement? *Learn. Instr.* 52 130–138. 10.1016/j.learninstruc.2017.05.007

[B84] Undheim A. M. Wichstrøm L. Sund A. M. (2011). Emotional and behavioral problems among school adolescents with and without reading difficulties as measured by the youth self-report: a one-year follow-up study. *Scand. J. Educ. Res.* 55 291–305. 10.1080/00313831.2011.576879

[B85] Valera-Pozo M. Adrover-Roig D. Pérez-Castelló J. A. Sanchez-Azanza V. A. Aguilar-Mediavilla E. (2020). Behavioral, emotional and school adjustment in adolescents with and without developmental language disorder (DLD) is related to family involvement. *Int. J. Environ. Res. Public Health* 17:1949. 10.3390/ijerph17061949 32188170 PMC7142754

[B86] Van den Bedem N. P. Dockrell J. E. van Alphen P. M. Kalicharan S. V. Rieffe C. (2018a). Victimization, bullying, and emotional competence: longitudinal associations in (Pre)adolescents with and without developmental language disorder. *J. Speech, Lang. Hear. Res.* 61 2028–2044. 10.1044/2018_JSLHR-L-17-0429 29998317

[B87] Van den Bedem N. P. Dockrell J. E. van Alphen P. M. Rooij M. Samson A. C. Harjunen E. L. (2018b). Depressive symptoms and emotion regulation strategies in children with and without developmental language disorder: a longitudinal study. *Int. J. Lang. Commun. Disord.* 53 1110–1123. 10.1111/1460-6984.12423 30141224 PMC6282613

[B88] van der Wilt F. van der Veen C. van Kruistum C. van Oers B. (2018). Why can’t I join? Peer rejection in early childhood education and the role of oral communicative competence. *Contemp. Educ. Psychol.* 54 247–254. 10.1016/j.cedpsych.2018.06.007

[B89] Wadman R. Durkin K. Conti-Ramsden G. (2011). Social stress in young people with specific language impairment. *J. Adolesc.* 34 421–431. 10.1016/j.adolescence.2010.06.010 20650511

[B90] Woodman A. C. (2014). Trajectories of stress among parents of children with disabilities: a dyadic analysis. *Fam. Relat.* 63:12049. 10.1111/fare.12049

[B91] Yildiz M. Yildirim K. Ates S. Rasinski T. (2012). Perceptions of Turkish parents with children identified as dyslexic about the problems that they and their children experience. *Read. Psychol.* 33 399–422. 10.1080/02702711.2010.515907

